# The Inositol Phosphatase SHIP-1 Inhibits NOD2-Induced NF-κB Activation by Disturbing the Interaction of XIAP with RIP2

**DOI:** 10.1371/journal.pone.0041005

**Published:** 2012-07-17

**Authors:** Claude Condé, Xavier Rambout, Marielle Lebrun, Aurore Lecat, Emmanuel Di Valentin, Franck Dequiedt, Jacques Piette, Geoffrey Gloire, Sylvie Legrand

**Affiliations:** 1 Laboratory of Virology and Immunology, Signal Transduction Unit, GIGA-R, University of Liège, Liège, Belgium; 2 The laboratory of protein signaling and interactions, Signal Transduction Unit, GIGA-R, University of Liège, Liège, Belgium; 3 GIGA – viral vector platform, GIGA-R, University of Liège, Liège, Belgium; 4 Interface Entreprises-Université Liège Science park, Liège, Belgium; McGill University, Canada

## Abstract

SHIP-1 is an inositol phosphatase predominantly expressed in hematopoietic cells. Over the ten past years, SHIP-1 has been described as an important regulator of immune functions. Here, we characterize a new inhibitory function for SHIP-1 in NOD2 signaling. NOD2 is a crucial cytoplasmic bacterial sensor that activates proinflammatory and antimicrobial responses upon bacterial invasion. We observed that SHIP-1 decreases NOD2-induced NF-κB activation in macrophages. This negative regulation relies on its interaction with XIAP. Indeed, we observed that XIAP is an essential mediator of the NOD2 signaling pathway that enables proper NF-κB activation in macrophages. Upon NOD2 activation, SHIP-1 C-terminal proline rich domain (PRD) interacts with XIAP, thereby disturbing the interaction between XIAP and RIP2 in order to decrease NF-κB signaling**.**

## Introduction

Innate immunity constitutes the first line of host defence against pathogens. It activates inflammation and initial antimicrobial responses prior to the onset of adaptive immunity. Recognition of invading pathogens is a crucial mechanism that relies on recognition of pathogen-associated molecular patterns (PAMPs) by patterns recognition receptors (PRRs). The PRR armada is composed of the membrane-associated toll-like receptors (TLRs, reviewed in [1]) that sense pathogens at cell surface and within the endosomes whereas the cytosolic NOD-like receptors (NLRs, reviewed in [2]) guard the intracellular compartment. NLRs, such as NOD1 and NOD2, are able to induce cytokines, chemokines and antimicrobial peptides production by activating the transcription factor nuclear factor-κB (NF-κB) and mitogen-activated protein kinases (MAPKs) [2]. NOD2 detects muramyl dipeptide (MDP) derived from peptidoglycan of both Gram positive and Gram negative bacteria, whereas NOD1 detect the tri-DAP (L-alanine – γ-D-Glutamic acid – *meso-*diaminopimelic acid), a structure mainly found in Gram-negative bacteria [3], [4]. Essential role for NOD2 in intestinal mucosa immunity is highlighted by the fact that mutations in the NOD2 gene are associated with increased risk to develop Crohn’s disease, an inflammatory disorder characterized by chronic inflammation of the gastrointestinal tract [5], [6], [7].

Structurally, NOD1 and NOD2 are composed of multiple leucine rich repeats (LRR) in the carboxyterminus, which mediate the recognition of their respective ligand and of a central nucleotide binding and oligomerization domain (NOD) that enables oligomerization upon activation. Finally, the N-terminal region of NOD1 and NOD2 encompasses one or two caspase recruitment domains (CARD), respectively, and mediates the recruitment of downstream effectors [8]. Upon activation by their ligand, NOD1 and NOD2 oligomerize and form an active platform, called the NODosome, which recruits downstream signaling proteins such as RIP2, a serine/threonine and tyrosine kinase. RIP2 autophosphorylates and is K63-polyubiquitinated by the cellular inhibitors of apoptosis 1 and 2 (cIAP1 and cIAP2) [9], thereby recruiting NEMO (also called IKKγ, IκB kinase γ) [10] and TAK1– TAB1/2/3 complex [9], [11], [12]. On one hand, TAK1 (TGF-β activated kinase-1) activates MAPKs and, on the other hand, it phosphorylates IKKβ, a subunit of the IκB kinase (IKK) complex composed of the regulatory subunit NEMO and the two catalytic subunits IKKα and IKKβ [13]. Activation of the IKK complex leads to the phosphorylation of inhibitory κB proteins, such as IκBα, that sequester the NF-κB in the cytoplasm. Phosphorylation of IκB proteins on serines 32 and 36 promotes their proteasomal degradation and results in release of NF-κB, which translocates into the nucleus where it regulates gene transcription [13].

IAP proteins (inhibitor of apoptosis) such as cIAP1, cIAP2 and XIAP are an evolutionarily conserved family of proteins first characterized as potent regulator of programmed cell death in various species ranging from insects to humans [14]. Structurally, IAP proteins possess several BIR domains (baculovirus IAP repeats) that mediate binding to caspases. Furthermore, in human, cIAP1, cIAP2 and XIAP were identified as potent apoptosis suppressors. Strikingly, XIAP, cIAP1 and cIAP2 contain a C-terminal RING domain that provides them with an ubiquitin E3 ligase activity and are implicated in other cellular signaling pathways than apoptosis [15], [16]. Among these, accumulating evidences suggest an important role for XIAP in NF-κB activation. For instance, XIAP was shown to interact with TAB1 *via* its BIR1 domain. This activates the TAK1 complex and promotes NF-κB activation [15], [17], [18]. Moreover, several recent studies have highlighted the role of XIAP in immune signaling since XIAP has been shown to interact with RIP2, thereby facilitating NOD2-induced NF-κB activation [19]. Furthermore, XIAP-deficient mice are more susceptible to *Listeria monocytogenes* infection compared to their WT littermates. These mice exhibit a dramatic reduction of NF-κB activation along with a decrease of proinflammatory cytokines production in response to *Listeria* infection [20]. These results strongly suggest an important role for XIAP in immune pathways.

SHIP-1 is an SH2-containing inositol 5′-phosphatase principally expressed by hematopoietic cells. SHIP-1 hydrolyses phosphatidylinositol triphosphate (PI-3,4,5-P3 or PIP3) and generates phosphatidylinositol biphosphate (PI-3,4-P2), thereby antagonizing PI3K signalization pathway and downmodulating cell proliferation, differentiation and survival [21], [22], [23]. SHIP-1 is composed of 3 domains: a central catalytic domain surrounded by a SH2 domain in the N-terminal part, and by a proline rich domain (PRD) and two phosphorylable tyrosines in the carboxyterminus. SH2 and PRD domains mediate interactions with other proteins, which dictate SHIP-1 biological functions. SHIP-1 is well characterized as a negative regulator of immune pathways (reviewed in [24]). Indeed, SHIP-1 decreases activation of the B cell receptor (BCR) after FcγRIIB engagement in B cells [25], [26], [27], [28], it downregulates CD16-mediated cytotoxicity in NK cells [29], [30] and degranulation of mast cells [31], [32], [33]. Strikingly, SHIP-1 is also implicated in downmodulation of TLR signaling. SHIP-1 KO macrophages exhibit an increased cytokines production in response to TLR4 triggering [34] and produce more interferon β (IFNβ) in response to TLR3 activation than their WT counterpart [35]. Altogether, these data show that SHIP-1 plays an important role in regulating TLRs pathway. Considering that TLR and NLR are related receptors sharing signaling components, we hypothesized that SHIP-1 could also downmodulate NLR activation pathways.

Here, we demonstrated that SHIP-1 is a negative regulator of NOD1 and NOD2-induced NF-κB activation. Indeed, we observed that the depletion of SHIP-1 specifically increases NOD1 and NOD2-dependent NF-κB activity. We demonstrated that the inhibitory capacity of SHIP-1 is not linked to its catalytic activity but relies on its PRD domain. A yeast two-hybrid screen revealed that SHIP-1 PRD region interacts with XIAP, which was recently described as intermediate in NOD2 pathway [9], [19]. In this study, we further confirmed that XIAP is essential to activate NF-κB in the course of NOD2 signaling and we also highlighted the crucial role of XIAP in NOD1 signaling since XIAP depletion in macrophages is associated with a dramatic decrease of NF-κB activation after NOD1 engagement. Mechanistically, we observed that, after NOD2 activation, SHIP-1 interacts with XIAP and disturbs the association of XIAP with RIP2, thereby decreasing NF-κB activation. Altogether, these results highlight a new negative regulator role for SHIP-1 during NOD1 and NOD2 signaling mediated by its interaction with XIAP.

## Results

### SHIP-1 Downregulates NOD2-induced NF-κB Activation

**Figure 1:**
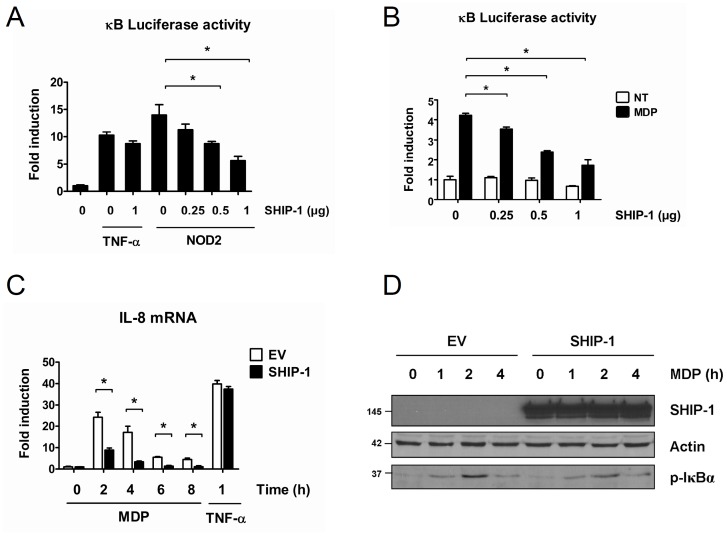
SHIP-1 decreases NOD2-induced NF-κB activation in epithelial cells. **A.** HEK293T cells were transfected with plasmids encoding a NF-κB luciferase reporter gene and NOD2 along with increasing amounts of SHIP-1. Positive control was achieved by treating cells transfected with SHIP-1 or with an empty vector with TNF-α (250 U/mL) for 6 h. NF-κB activity was measured by luciferase assay 36 h post transfection (mean ± S.D (n = 3), *p<0.01). **B.** GNV cells were transfected with a NF-κB luciferase reporter gene along with increasing amounts of SHIP-1. Cells were subsequently treated with MDP (1 µg/mL) for 18 h or left untreated. NF-κB activity was determined by luciferase assay (mean ± S.D (n = 3), *p<0.04). **C.** GNV cells were transfected with 2.5 µg of either a SHIP-1 or an empty vector. 24 h post transfection, cells were treated with MDP (50 µg/mL) for indicated periods or with TNF-α (250 U/mL) for 1 h. RNA was extracted and *il-8* mRNA level was measured by quantitative real-time PCR (mean ± S.D (n = 3), *p<0.001). **D.** GNV cells were transfected with 2.5 µg of either a SHIP-1 or an empty vector. 24 h post transfection, cells were treated with MDP (50 µg/mL) for indicated periods and proteins were extracted. Samples were immunoblotted with an anti-SHIP-1 and an anti-p-IκBα antibody. Actin was used as loading control.

SHIP-1 is mainly expressed by hematopoietic cells where negative regulation of immune pathways by SHIP-1 is commonly described [25], [26], [28], [30], [36]. For instance, in macrophages, SHIP-1 decreases the activation of TLR3 and TLR4, two members of the PRR family [34], [35]. We assumed that SHIP-1 could also downregulate other PRRs, such as NLRs. Consequently, we investigated the impact of SHIP-1 on NOD2 signaling pathway. We first used human embryonic kidney cells, *i.e.* HEK293T cells, which express neither SHIP-1 nor NOD2 and where a strong NF-κB activity can be observed following NOD2 overexpression [11]. We monitored NF-κB activity by luciferase gene reporter assay in HEK293T cells overexpressing NOD2 along with or without increasing amounts of SHIP-1. We observed that overexpression of SHIP-1 decreases NOD2-induced NF-κB activation in a dose-dependent manner (Fig 1A). Interestingly, we did not observe any impact of SHIP-1 expression on TNF-α-mediated NF-κB activation, thereby showing that SHIP-1 is not a general NF-κB inhibitor but seems to be specific of the NOD2 pathway.

To better characterize the negative regulator role of SHIP-1 on the NOD2 pathway, we used HEK293 cells stably expressing a NOD2 transgene, called GNV cells. These cells do not express SHIP-1 endogenously and are responsive to muramyl dipeptide (MDP), the natural ligand of NOD2 (Lecat et al., in prep.). These cells were transfected with increasing amounts of SHIP-1 or with an empty vector, treated with MDP and the NF-κB activation was subsequently measured by luciferase reporter gene assay (Fig 1B). We observed that, in SHIP-1 expressing cells, the NF-κB activity induced by MDP is dramatically reduced compared to empty vector-transfected cells. Furthermore, SHIP-1 decreases MDP-induced NF-κB activity in a dose-dependent manner. We also evaluated NOD2-induced NF-κB activation by analysing the transcription level of *il-8*, an NF-κB-dependent gene induced by NOD2 activation [11]. We observed that GNV cells expressing SHIP-1 exhibit a dramatic reduction of *il-8* transcription in response to MDP treatment (Fig 1C). Again, no effect of SHIP-1 was observed on TNF-α-induced *il-8* transcription, thereby confirming the specificity of SHIP-1 for the NOD2 pathway. We next monitored the phosphorylation of IκBα on serine 32 and 36, which are phosphorylated by the IKK complex during NF-κB activation. We observed that SHIP-1-expressing cells exhibit a decreased IκBα phosphorylation upon MDP treatment compared to empty vector-transfected cells (Fig 1D).

**Figure 2:**
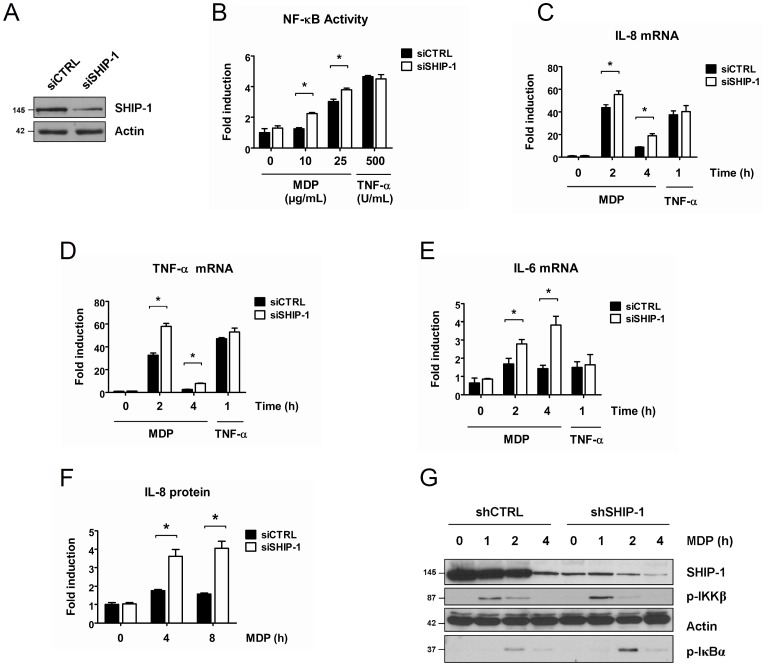
SHIP-1 decreases NOD2-induced NF-κB activation in monocytes/macrophages. **A.** THP1-Xblue cells were transfected with a siRNA targeting SHIP-1 (siSHIP-1) or with a non-target siRNA (siCTRL). 48 h post transfection, proteins were extracted. Samples were immunoblotted with an anti-SHIP-1 antibody. Actin was used as a loading control. **B.** THP1-Xblue cells were transfected with a siRNA targeting SHIP-1 (siSHIP-1) or with a non-target siRNA (siCTRL). 36 h post transfection, cells were treated with MDP (10 or 25 µg/mL) or TNF-α (500 U/mL) for 16 h or left untreated. NF-κB activity was measured by colorimetric enzyme assay (mean ± S.D (n = 3), *p<0.003). **C,D, E.** THP1-Xblue cells were transfected with a siRNA targeting SHIP-1 (siSHIP-1) or with a non-target siRNA (siCTRL). 48 h after transfection, cells were treated with MDP (100 µg/mL) or with TNF-α (500 U/mL) for indicated period. RNAs were extracted and *il-8, il-6 and tnf-α* mRNA levels were measured by quantitative real-time PCR (Mean ± S.D (n = 3), *p<0.0001). **F.** THP1 cells were transfected with a siRNA targeting SHIP-1 (siSHIP-1) or with a non-target siRNA (siCTRL). 48 h after transfection, cells were treated with MDP (100 µg/mL) for indicated period. Cell supernatant was harvested and IL-8 protein content was determined by ELISA. **G.** THP1 cells stably expressing a shRNA targeting SHIP-1 (shSHIP-1) or a non target shRNA (shCTRL) were treated with MDP (100 µg/mL) for indicated periods. Proteins were extracted and samples were immunoblotted with an anti-p-IKKβ, an anti-p-IκBα and an anti-SHIP-1 antibody, respectively. Actin was used as a loading control.

To complement the results obtained in overexpression experiments, we selected a human monocytic cell line, *i.e.* THP1 cells. These immune cells express endogenously both SHIP-1 and NOD2. In some experiments, we also used the THP1-Xblue cells. These THP1 cells are stably transfected with a reporter construct expressing the secreted embryonic alkaline phosphatase (SEAP) under the control of an NF-κB-dependent promoter. This reporter protein is readily measurable in the culture supernatant by colorimetric assay. First, we downmodulated SHIP-1 expression by siRNA in THP1-Xblue cells. As a control, cells were transfected with a non-target siRNA (Fig 2A). We observed that SHIP-1-silenced THP1-Xblue cells exhibit an increased NF-κB activation after MDP exposure (Fig 2B). In addition, we also monitored NF-κB activation by analysing the transcription levels of NF-κB-dependent genes: *il-8*, a chemokine, and of *il-6* and *tnf-α*, two proinflammatory cytokines, whose transcription is induced upon NOD2 stimulation. We observed that *il-8, il-6 and tnf-α* mRNA transcription greatly increases in SHIP-1-silenced cells stimulated with MDP compared to the siCTRL-transfected cells (Fig 2C, 2D, 2E). Of note, we confirmed that silencing of SHIP-1 does not affect TNF-R signaling as we observed no difference of *il-8, il-6 and tnf-α* mRNA levels between SHIP-1-silenced cells and control cells (Fig 2C, 2D, 2E). We next verified that this increased genes transcription correlates with an increased protein release by analysing the IL-8 secretion in the extracellular medium. We observed an enhanced IL-8 secretion in SHIP-1-silenced cells stimulated with MDP (Fig 2F). Finally, we also generated THP1 cells stably expressing a shRNA targeting SHIP-1 in order to study the effect of SHIP-1 on earlier steps of NOD2-mediated NF-κB activation such as IKKβ and IκBα phosphorylation, two hallmarks of the IKK complex activation. As a control, cells were transduced with a non-target shRNA. We observed that silencing of SHIP-1 leads to a transient increased phosphorylation of IKKβ, which likely translates an enhanced activity of the IKK complex when SHIP-1 is depleted (Fig 2G). Accordingly, we also observed that IκBα phosphorylation is increased in SHIP-1-silenced cells (Fig 2G).

Altogether, these data confirm that SHIP-1 decreases NF-κB activation upon NOD2 engagement in THP1 cells.

### SHIP-1 Interacts with XIAP via Association of its PRD Domain with the BIR2 Domain of XIAP

In order to understand how SHIP-1 downregulates NOD2 signaling pathway, we performed a yeast two-hybrid (Y2H) screen to identify new interaction partners of SHIP-1. As a bait, we used either full length or truncated versions of SHIP-1 (one containing the SH2 domain and the other encompassing the PRD region), whereas the prey was constituted of the hORFeome 5.1 [37]. This Y2H screen enables the identification of two well known SHIP-1 interacting proteins, *i.e.* CIN85 and DAB1 [38], [39]. The screen identified XIAP as interacting with the PRD domain of SHIP-1. Interestingly, XIAP is a member of the IAP family that has been recently identified as an intermediate of NOD2 signaling [19].

We first confirmed the Y2H results by systematically verifying all interaction candidates. To this end, we used haploid yeast cells of opposite mating type, each expressing either the different SHIP-1 constructs or the identified interactants. These haploid yeasts were mated according to the interacting pairs identified in the original screen and were tested for reproducible Y2H phenotypes in order to verify reporter gene activation. This experiment allows us to confirm the interaction of the PRD domain of SHIP-1 with XIAP (data not shown).

**Figure 3:**
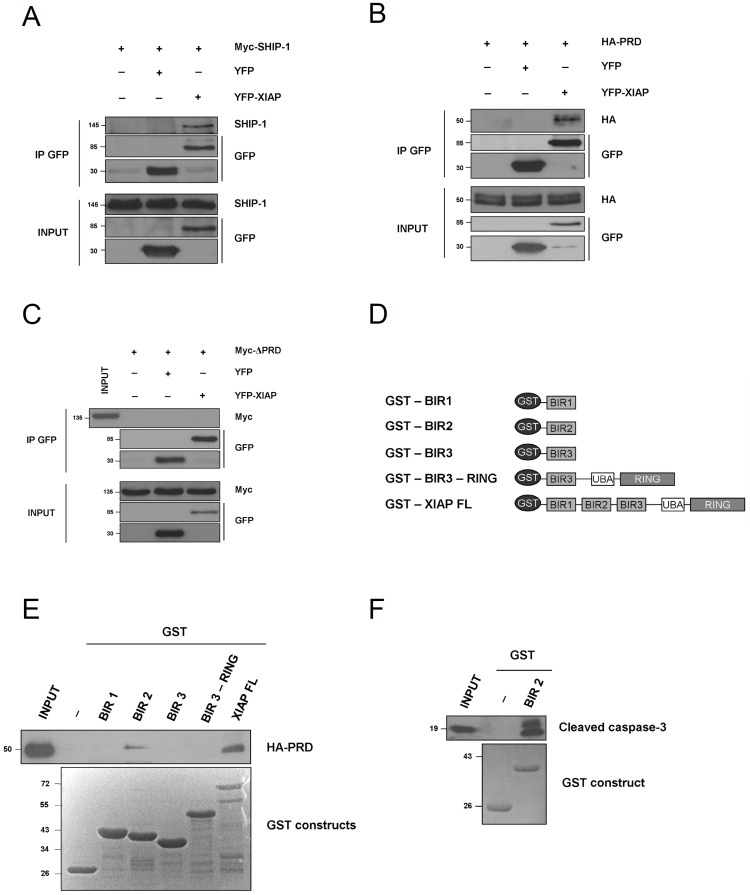
SHIP-1 interacts with XIAP *via* its PRD domain. **A.** HEK293T cells were transfected with SHIP-1 alone or along with YFP and YFP-XIAP, respectively. 24 h post transfection, proteins were extracted. YFP-fused XIAP was immunoprecipitated with anti-GFP antibody and coimmunoprecipitated SHIP-1 was revealed by immunoblotting with an anti-SHIP-1 antibody. **B.** HEK293T cells were transfected with HA-PRD construct alone or along with YFP and YFP-XIAP, respectively. 24 h post transfection, proteins were extracted. YFP-fused XIAP was immunoprecipitated with an anti-GFP antibody and coimmunoprecipitated HA-PRD was revealed by immunoblotting with an anti-HA antibody. **C.** HEK293T cells were transfected with Myc-ΔPRD construct alone or along with YFP and YFP-XIAP, respectively. 24 h post transfection, proteins were extracted. YFP-fused XIAP was immunoprecipitated with GFP-antibody and coimmunoprecipitated Myc-ΔPRD was revealed by immunoblotting with an anti-Myc antibody. **D.** Schematic representation of GST-XIAP constructions. **E.** Lysates of HEK293T transfected with HA-PRD construct were incubated with 200 µg of various recombinant GST-XIAP constructs or with GST alone. Coimmunoprecipitated SHIP-1 was revealed by immunoblotting with an anti-SHIP-1 antibody. **F.** Lysates of HEK293T previously incubated with cytochrome *c* and dATP for 30 min at 37°C were incubated with 200 µg of GST-BIR2 or GST alone. Coimmunoprecipitated cleaved caspase-3 was revealed by immunoblotting with an anti-cleaved caspase-3 antibody.

To further validate the Y2H experiment, we carried out coimmunoprecipation experiments in HEK293T cells transfected with a SHIP-1 encoding vector along with an YFP-XIAP construct or with YFP alone. The YFP proteins were immunoprecipitated with a GFP antibody that crossreacted with the YFP protein. We observed that SHIP-1 coimmunoprecipitates with YFP-XIAP but not with YFP protein (Fig 3A). Since we have identified the PRD domain of SHIP-1 as the interacting region with XIAP in the Y2H screen, we constructed a truncated version of SHIP-1 that only contains this PRD region fused to the HA tag (called HA-PRD) and tested its interaction with XIAP. As expected, we observed a coimmunoprecipitation of this HA-PRD construct with the YFP-XIAP whereas no interaction between HA-PRD and the YFP protein was observed (Fig 3B). Finally, we also generated a truncation mutant of SHIP-1 lacking the PRD domain and fused to a Myc tag, called Myc-ΔPRD. Interestingly, we failed to observe any interaction between this ΔPRD mutant and XIAP (Fig 3C).

To better define the interaction between XIAP and SHIP-1, we also performed a GST pull down experiment using GST-XIAP constructs encoding either the full length protein (FL) or truncated versions of XIAP (Fig 3D). HEK293T cells transfected with the HA-PRD construct described above were lysed and incubated with the recombinant GST constructs immobilized on glutathione-sepharose beads. We observed a pull down of HA-PRD with the GST-XIAP FL but also with the GST- BIR2 construct (Fig 3E). As a control, we monitored the interaction between the GST-BIR2 and the cleaved caspase-3 (Fig 3F), since interaction between BIR2 domain of XIAP and cleaved caspase-3 has been extensively described [40], [41], [42].

In conclusion, these results indicate that SHIP-1 PRD region interacts with the BIR2 domain of XIAP.

### XIAP is an Essential and Specific Intermediate during NOD2-induced NF-κB Activation in Macrophages

**Figure 4:**
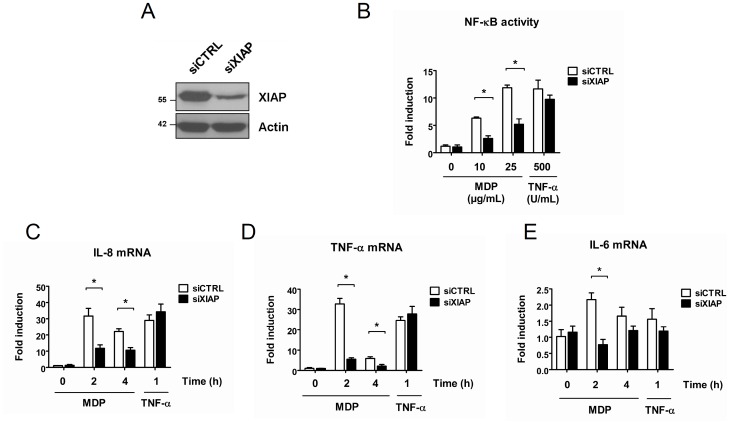
XIAP is an essential and specific intermediate of NOD2-induced NF-κB activation. **A.** THP1-Xblue cells were transfected with a siRNA targeting XIAP (siXIAP) or with a non-target siRNA (siCTRL). 48 h post transfection, proteins were extracted. Samples were immunoblotted with an anti-XIAP antibody. Actin was used as a loading control. **B.** THP1-Xblue cells were transfected with a siRNA targeting XIAP (siXIAP) or with a non-target siRNA (siCTRL). 36 h post infection, cells were treated for 16 h with MDP (10 or 25 µg/mL) or with TNF-α (500 U/mL) or left untreated. NF-κB activity was measured by colorimetric enzyme assay (mean ± S.D (n = 3), *p<0.004). **C, D, E.** THP1-Xblue cells were transfected with a siRNA targeting XIAP (siXIAP) or with a scramble siRNA (siCTRL). 48 h post transfection, cells were treated with MDP (100 µg/mL) or with TNF-α (500 U/mL) for indicated periods. RNAs were extracted and *il-8, il-6 and tnf-α* mRNA levels were measured by quantitative real-time PCR (Mean ± S.D (n = 3), *p<0.0001).

Recently, XIAP has been reported to play a key role in NOD2 signaling at least in epithelial cells. First, XIAP was shown to interact with the RIP2 – NOD2 complex [19]. Considering that XIAP interacts also with the TAK1 – TAB1/2 complex via its BIR1 domain [15], [17], it is speculated that XIAP acts as a scaffold protein enabling the recruitment of TAK1 – TAB1/2 complex to the NOD2 – RIP2 complex in order to induce the proper IKK complex activation [19]. In this regard, XIAP-deficient epithelial cells are unable to activate NF-κB and to induce *il-8* gene transcription upon MDP stimulation [19]. Furthermore, bone marrow-derived macrophages (BMDMs) from XIAP KO mice exhibit a decrease of IL-6 production in response to MDP treatment [20]. Altogether, these reports suggest that XIAP is necessary to activate NF-κB during NOD2 signaling. As we used the human monocytic THP1 cells, we verified whether XIAP is also essential for NOD2 signaling in our cellular model by downmodulating XIAP expression in THP1-Xblue cells (Fig 4A) and subsequently measuring NF-κB activation. As a control, cells were transfected with a non-target siRNA. We observed that the depletion of XIAP in macrophages leads to a dramatic decrease of NF-κB activation after MDP exposure (Fig 4B). Moreover, MDP-induced *il-8, il-6 and tnf-α* transcription was greatly reduced in XIAP-silenced macrophages (Fig 4C, 4D, 4E). Of note, we observed that silencing of XIAP did not affect TNF-R signaling (Fig 4B, 4C, 4D, 4E), since no difference between siCTRL- and siXIAP-transfected cells could be observed both in colorimetric assay and in quantitative real-time PCR, indicating that XIAP is not required for TNF-R signaling. These results are in accordance with previous data obtained in epithelial cells [19] and highlight the crucial and specific role of XIAP during NOD2 signaling in our cell model.

### SHIP-1 Interacts with XIAP during NOD2 Stimulation in Macrophages

**Figure 5:**
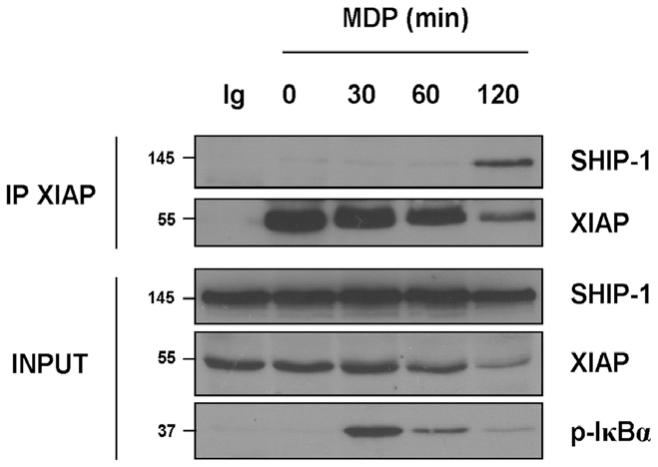
SHIP-1 interacts with XIAP in the course of NOD2 signaling. THP1 cells were treated with MDP (100 µg/mL) for indicated periods and proteins were extracted. Samples were immunoprecipitated with an anti-XIAP antibody or with an IgG control (Ig). Coimmunoprecipitated SHIP-1 was revealed by immunoblotting with an anti-SHIP-1 antibody. Expression of different proteins in the whole cell lysate (INPUT) was verified by immunoblotting with anti-XIAP, anti-SHIP-1 and anti-p-IκBα antibodies, respectively.

As SHIP-1 has been shown to interact with XIAP, and because both proteins are implicated in NOD2 signaling, we tried to observe an endogenous coimmunoprecipitation between SHIP-1 and XIAP in THP-1 monocytic cells in the course of NOD2 signaling. We performed a kinetic of treatment with MDP and subsequently immunoprecipitated XIAP. In unstimulated cells, we failed to observe any coimmunoprecipitation between SHIP-1 and XIAP. However, two hours after MDP stimulation, we detected SHIP-1 in XIAP immunoprecipitate (Fig 5). Strikingly, this interaction takes place when the phosphorylation of IκBα decreases, indicating that SHIP-1 and XIAP interact during the downmodulation phase of NF-κB signaling. Strikingly, XIAP seems to be modified or degraded at longer time point during MDP treatment (Fig 5).

### SHIP-1 PRD Region is Necessary and Sufficient to Decrease NOD2-induced NF-κB Activity

**Figure 6:**
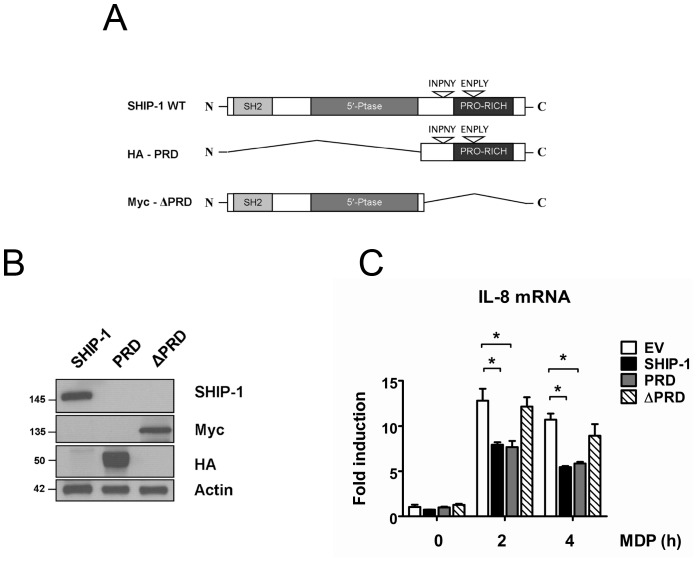
SHIP-1 PRD region is necessary and sufficient to decrease NOD2-induced NF-κB activity. **A.** Schematic representation of SHIP-1 deletion constructs. **B.** GNV cells were transfected with 2.5 µg of SHIP-1, HA-PRD (PRD), Myc-ΔPRD (ΔPRD) encoding vectors. 24 h post transfection, proteins were extracted. Samples were immunoblotted with an anti-SHIP-1, an anti-HA or an anti-Myc antibody. Actin was used as a loading control. **C.** GNV cells were transfected with 2.5 µg of either a SHIP-1 (SHIP-1) or a HA-PRD (PRD) or a Myc-ΔPRD (ΔPRD) or an empty vector (EV). 24 h post transfection, cells were treated with MDP (50 µg/mL) for indicated periods. RNAs were extracted and *il-8* mRNA level was measured by quantitative real-time PCR (mean ± S.D (n = 3), *p<0.004).

Because we observed that the PRD domain of SHIP-1 interacts with XIAP, we wondered whether this domain of SHIP-1 is required to inhibit NOD2-induced NF-κB signaling. Therefore, we used 3 constructs: the SHIP-1 full length protein (SHIP-1 WT), the HA-PRD construct and the Myc-ΔPRD deletion mutant (Fig 6A). We verified that the three proteins were properly expressed in HEK293T cells (Fig 6B). To compare inhibitory properties of these three SHIP-1 proteins on NOD2-induced NF-κB signaling, we monitored *il-8* mRNA levels in GNV cells following overexpression of each construct and MDP treatment. We observed that GNV cells expressing either SHIP-1 or the isolated PRD domain exhibit a decreased *il-8* gene transcription in response to MDP treatment. In contrast, cells expressing the Myc-ΔPRD deletion construct exhibit similar *il-8* mRNA levels than cells transfected with an empty vector (Fig 6C). These results indicate that the SHIP-1 PRD domain, which mediates the interaction with XIAP, is essential to decrease NOD2-induced NF-κB activation. In addition, enzymatic activity of SHIP-1 is not required for NF-κB inhibition since the HA-PRD construct, which lacks the entire catalytic domain, is sufficient to reduce the MDP-induced NF-κB activity. These observations sustain our hypothesis that SHIP-1 inhibits the NOD2 pathway by interacting with XIAP *via* its PRD domain. We assume that SHIP-1 should modify somehow XIAP functions to decrease NOD2 activation.

### SHIP-1 Decreases NOD1-induced NF-κB Activation

**Figure 7:**
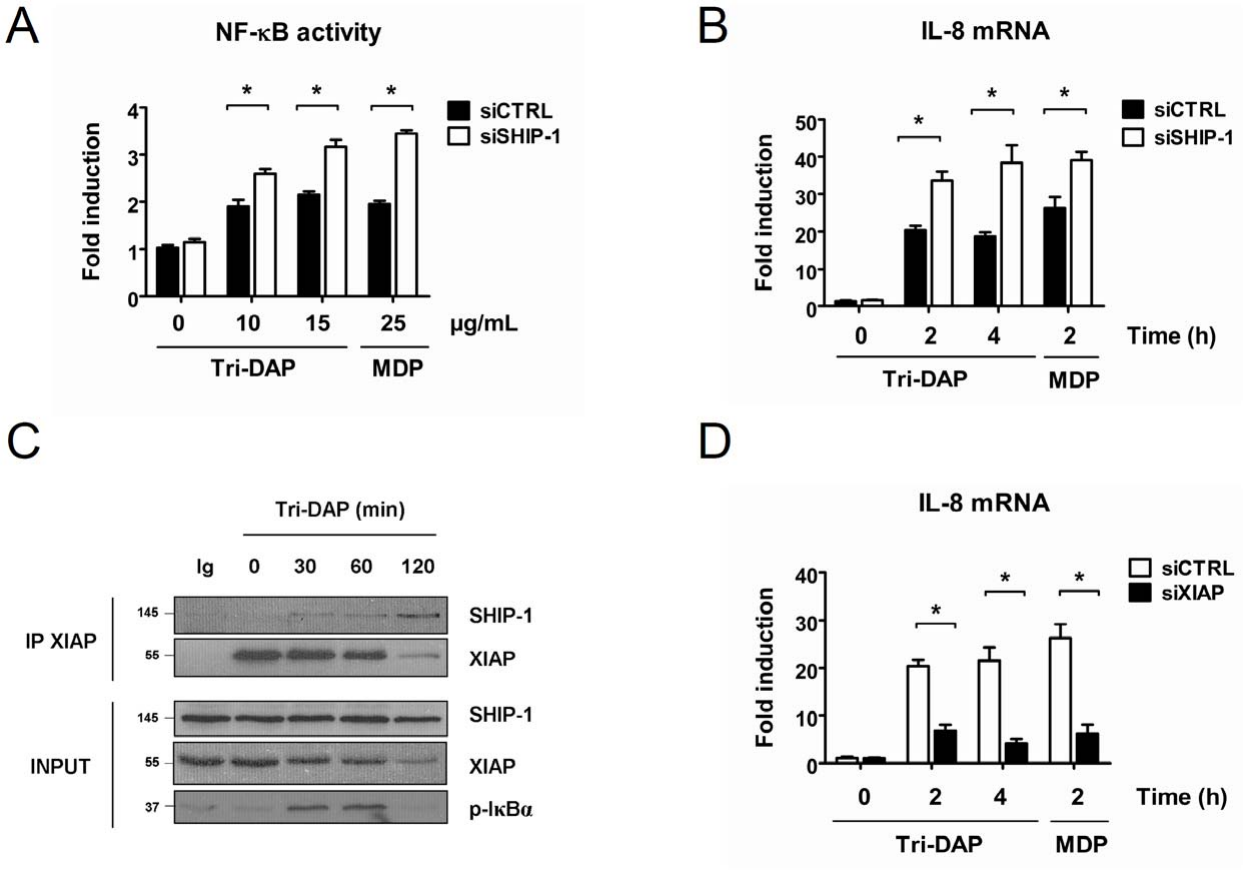
SHIP-1 decreases NOD1-induced NF-κB activation in monocytes/macrophages and interacts with XIAP in the course of NOD1 signaling. **A.** THP1-Xblue cells were transfected with a siRNA targeting SHIP-1 (siSHIP-1) or with a non-target siRNA (siCTRL). 36 h post transfection, cells were treated with Tri-DAP (10 or 15 µg/mL) or MDP (25 µg/mL) for 16 h or left untreated. NF-κB activity was measured by colorimetric enzyme assay (mean ± S.D (n = 3), *p<0.003). **B.** THP1-Xblue cells were transfected with a siRNA targeting SHIP-1 (siSHIP-1) or with a non-target siRNA (siCTRL). 48 h after transfection, cells were treated with Tri-DAP (75 µg/mL) or with MDP (100 µg/mL) for indicated periods. RNAs were extracted and *il-8* mRNA level was measured by quantitative real-time PCR (Mean ± S.D (n = 3), *p<0.0001). **C.** THP1 cells were treated with Tri-DAP (75 µg/mL) for indicated periods and proteins were extracted. Samples were immunoprecipitated with an anti-XIAP antibody or with an IgG control (Ig). Coimmunoprecipitated SHIP-1 was revealed by immunoblotting with an anti-SHIP-1 antibody. Expression of different proteins in the whole cell lysate (INPUT) was verified by immunoblotting with anti-XIAP, anti-SHIP-1 and anti-p-IκBα antibodies, respectively. **D.** THP1-Xblue cells were transfected with a siRNA targeting XIAP (siXIAP) or with a scramble siRNA (siCTRL). 48 h post transfection, cells were treated with Tri-DAP (75 µg/mL) or MDP (100 µg/mL) for indicated periods. RNAs were extracted and *il-8* mRNA level was measured by quantitative real-time PCR (Mean ± S.D (n = 3), *p<0.0001).

Considering the close proximity between NOD1 and NOD2 signaling pathways, we wondered whether SHIP-1 could also decrease NOD1-induced NF-κB activation. Therefore, we transfected the THP1-Xblue cells with a siRNA targeting SHIP-1. We analysed the NF-κB activity by colorimetric assay after exposure to Tri-DAP, the natural ligand of NOD1 [4]. We observed that SHIP-1-depleted cells exhibit an increased NF-κB activation in response to Tri-DAP stimulation, exactly as it has been shown upon MDP treatment (Fig 7A). We confirmed the NF-κB inhibitory capacity of SHIP-1 by analysing *il-8, il-6* and *tnf-α* mRNA levels upon NOD1 activation. We observed that transcription of these three genes was increased in SHIP-1-silenced cells after Tri-DAP exposure (Fig 7B and Fig S1).

As we had observed that SHIP-1 interacts with XIAP during NOD2 stimulation, we further checked whether SHIP-1 also associates with XIAP in the course of NOD1 signaling. Interestingly, an endogenous interaction between XIAP and SHIP-1 is also detected in Tri-DAP stimulated cells, whereas no interaction is detected in resting cells (Fig 7C). Again, this interaction takes place when the phosphorylation of IκBα decreases, indicating that SHIP-1 and XIAP interact during the downmodulation phase of NF-κB signaling. Strikingly, we also observed that XIAP seems to be modified or degraded at longer time point during Tri-DAP treatment.

We next assayed the role of XIAP in NOD1 signaling. As we and others have observed that XIAP is an essential intermediate of NOD2 signaling, we wondered whether XIAP is also required for NOD1 signaling. Therefore, we silenced XIAP by siRNA in THP1 cells. We subsequently analysed *il-8*, *il-6* and *tnf-α* mRNA levels. We observed that the transcription of these three genes in response to NOD1 activation is dramatically reduced in XIAP-silenced cells compared to siCTRL-transfected cells (Fig 7D and Fig S2). These results lead us to conclude that, firstly, SHIP-1 is also a downmodulator of NOD1-induced NF-κB activation. Secondly, we demonstrated that XIAP is required for the proper NF-κB activation in the course of NOD1 signaling, exactly as for NOD2 signaling. Finally, we demonstrated that SHIP-1 also interacts with XIAP after Tri-DAP stimulation thereby supporting the hypothesis that SHIP-1 interferes somehow with XIAP to decrease both NOD1 and NOD2 signaling.

### SHIP-1 Decreases XIAP and RIP2 Interaction, thereby Inhibiting NF-κB

**Figure 8:**
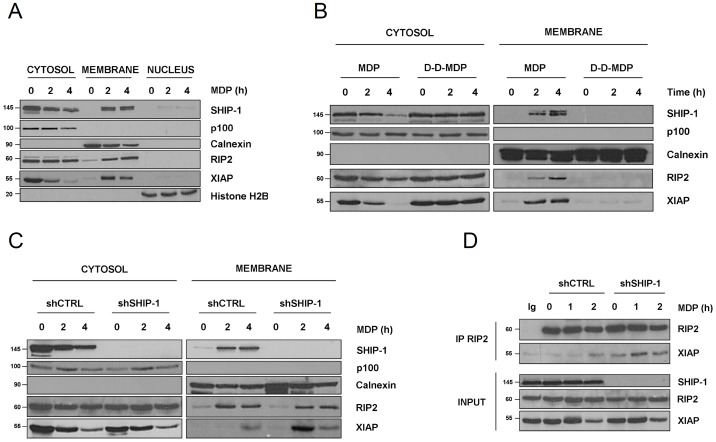
SHIP-1 decreases membrane recruitment of XIAP and interferes with the binding of XIAP to RIP2. **A.** THP1 cells were treated with MDP (100 µg/mL) for indicated periods. Cell fractionation was performed and equal amount of cytosolic, membrane and nuclear fractions were subjected to SDS-PAGE. Samples were immunoblotted with an anti-SHIP-1, an anti-XIAP and an anti-RIP2 antibody. Loading control and purity of cytosolic, membrane and nuclear fractions were assayed by immunoblotting p100, Calnexin and Histone H2B, respectively. **B.** THP1 cells were treated with MDP (100 µg/mL) or with an inactive isomer: D-D-MDP (100 µg/mL) for indicated periods and cell fractionation was performed. Cytosolic and membrane fractions were subjected to SDS-PAGE and samples were subsequently immunoblotted with an anti-SHIP-1, an anti-XIAP and an anti-RIP2 antibody. Loading control and purity of cytosolic and membrane fractions were assayed by immunoblotting p100 and Calnexin, respectively. **C.** THP1 cells stably expressing a non-target shRNA (shCTRL) or a shRNA targeting SHIP-1 (shSHIP-1) were treated with MDP (100 µg/mL) for indicated periods and cell fractionation was performed. Cytosolic and membrane fractions were subjected to SDS-PAGE and samples were subsequently immunoblotted with an anti-SHIP-1, an anti-XIAP and an anti-RIP2 antibody, respectively. Loading control and purity of cytosolic and membrane fractions were assayed by immunoblotting p100 and Calnexin, respectively. **D.** THP1 cells were treated with MDP (100 µg/mL) for indicated periods. RIP2 was subsequently immunoprecipitated and coimmunoprecipitated XIAP was revealed with an anti-XIAP antibody. Expression of different proteins in the whole cell lysate (INPUT) was verified by immunoblotting with anti-XIAP, anti-RIP2 and anti-SHIP-1 antibodies, respectively.

In long-time stimulated cells, either with MDP or Tri-DAP, we have observed by western blotting an apparent decrease of XIAP and SHIP-1 protein content (Fig 2G, Fig 5, Fig 7C). As the NODosome is well characterized to signal from the plasma membrane [43] and because proteins implicated in NOD1 and NOD2 signaling are recruited at the bacterial entry site [44], we wondered whether the apparent decrease of SHIP-1 and XIAP protein amount was due to a potential relocalization of the proteins to the membrane compartment, which is not correctly solubilised in our protein extraction. To determine whether NOD2 activation affects the localization of SHIP-1 and XIAP, we performed cell fractionation in order to isolate cytoplasmic, membrane and nuclear extracts. We observed that in unstimulated cells, SHIP-1, XIAP and RIP2 are found in the cytoplasmic extracts (Fig 8A). However, upon stimulation with MDP, we observed that XIAP, SHIP-1 and RIP2 gradually decrease in the cytoplasmic extracts, whereas they accumulate in the membrane fractions. The purity of the different fraction**s** was verified by analysing the localisation of p100, the strictly cytosolic precursor of p52, of Calnexin, a type I membrane protein and of the histone H2B, a nuclear-restricted protein (Fig 8A). Subsequently, we verified that SHIP-1, XIAP and RIP2 delocalization was due to NOD2 activation and does not rely on the entry of small peptides into the cell. Therefore, we used an inactive isomer of MDP, called D-D-MDP, where the L-alanine is substituted by a D-alanine. The resulting peptide fails to activate NOD2. Strikingly, RIP2 and XIAP failed to relocalize to the membrane compartment in cells treated with this inactive MDP isomer, whereas both proteins are recruited to the membrane fraction in cells treated with MDP (Fig 8B). This result demonstrated that RIP2 and XIAP are specifically recruited at the membrane during NOD2 activation.

We next wondered whether SHIP-1 affects the relocalization of RIP2 or XIAP. Therefore, we used the SHIP-1-silenced THP1 cells. We observed that in SHIP-1-depleted cells, XIAP translocates in a greater quantity in the membrane fraction, whereas the relocalization of RIP2 is not affected by SHIP-1 silencing (Fig 8C).

Recently, RIP2 has been described to interact with XIAP *via* its BIR2 domain [19]. This interaction is considered to facilitate TAK1 recruitment, thereby accelerating IKK complex activation. Since we have demonstrated that SHIP-1 interacts also with the BIR2 domain of XIAP, we hypothesized that SHIP-1 could alter the binding of XIAP to RIP2, thereby downmodulating NOD2-induced NF-κB activation. Therefore, we immunoprecipitated RIP2 and analysed XIAP coimmunoprecipitation. We observed that in control cells, an interaction between XIAP and RIP2 could only be detected after treatment with MDP. Strikingly, in SHIP-1-silenced cells, we observed that the interaction between XIAP and RIP2 is already detectable in unstimulated cells, and further increase in MDP-stimulated conditions to reach higher levels than in shCTRL cells.

Altogether these results point out that SHIP-1 interferes with the interaction of XIAP with RIP2. Moreover, SHIP-1 also decreases the recruitment of XIAP to the membrane compartment upon MDP stimulation.

## Discussion

NOD1 and NOD2 are crucial sensors of bacterial invasion and critical regulators of immune response. Moreover, mutations in NOD2 gene are associated with increased risks of Crohn’s disease (CD) development. In this study, we have identified SHIP-1 as a new downmodulator of both NOD1 and NOD2 pathways, at least in monocytic cells. SHIP-1 was already characterized as an inhibitor of TLR4 and TLR3 pathways. Interestingly, its inhibitory capacities in TLR pathways are most probably linked to its catalytic activity. At the opposite, we have demonstrated that, in NOD2 pathway, SHIP-1 decreases NF-κB activation by a mechanism that does not involve its catalytic properties but rather relies on its PRD domain. We demonstrated that SHIP-1 interacts with XIAP and disturbs both the membrane recruitment of XIAP and its interaction with RIP2 thereby downmodulating NOD1- and NOD2-induced NF-κB activation.

Considering the role of SHIP-1 in NOD2 signaling, it has been recently demonstrated that SHIP-1-deficient mice develop a Crohn’s disease (CD) like ileitis [45]. SHIP-1 KO mice exhibit intestinal injuries closely related to lesions observed in CD patients. On one hand, inflammation of the gastrointestinal tract of SHIP-1 KO mice is restricted to the ileum, a zone particularly affected in CD. On the other hand, SHIP-1^−/−^ mice exhibit ulcers infiltrated by neutrophils, thickening of the bowel wall and collagen fibrosis which are some of the hallmarks of CD [45], [46]. How NOD2 mutations prime development of CD is still under debate. The current hypothesis is that NOD2 mutations associated with development of CD result in a loss of function of NOD2. Indeed, NOD2 variants were shown to loose their ability to induce correct NF-κB activation and cytokines production in response to bacterial infection [7], [47], [48]. In our model, SHIP-1 downmodulates NOD2 pathway and therefore loss of SHIP-1 expression would be expected to decrease susceptibility to CD, however *in vivo* the opposite is observed. Nevertheless, we cannot exclude that deregulation of NOD2 pathway either by loss or gain of function could increase risk of CD development. Loss of SHIP-1 should result in an extended activation of NOD2 signaling and increased inflammation. Besides, CD is characterised by chronic inflammation of the intestinal tract and both NLR and TLR signaling pathways are implicated in the disease development. As SHIP-1 is a downmodulator of both NLR and TLR pathways, it is not surprising that SHIP-1 KO mice exhibited such an ileitis.

Previous studies demonstrate that XIAP is implicated in innate immune response. For instance, it has been observed that XIAP is essential to induce a proper NF-κB activation in murine macrophages in response to *Chlamydophila pneumoniae* infection, a Gram negative invasive bacterium [49]. Likewise, XIAP KO mice are more sensitive to *Listeria monocytogenes* infection. Moreover, XIAP-deficient cells are unable to activate NF-κB and to induce *il-8* gene transcription upon MDP stimulation [19]. Finally, BMDMs from XIAP KO mice exhibit a decrease of IL-6 production in response to MDP treatment [20]. According to these studies, we have confirmed that XIAP is required to properly activate NF-κB during NOD2 signaling in human monocytes/macrophages (Fig 4B, 4C, 4D and 4E). Moreover, we have demonstrated that XIAP is an essential intermediate in NOD1 signaling as well (Fig 7D). Mechanistically, we observed that SHIP-1 interacts with XIAP in the course of both NOD1 and NOD2 signaling, thereby disturbing the interaction between XIAP and RIP2 (Fig 5, 7C, 8D) and decreasing NF-κB activation (Fig 2F). As we observed that in SHIP-1-depleted cells, the interaction between XIAP and RIP2 increases, we speculated that SHIP-1 alters the binding of XIAP to RIP2. Recently, it has been demonstrated that XIAP interacts with RIP2 *via* its BIR2 domain [19]. Strikingly, in our pull down experiment, we established that the PRD domain of SHIP-1 interacts with the BIR2 domain of XIAP. As both SHIP-1 and RIP2 interact with the same domain of XIAP, it is tempting to speculate that, in the course of NOD1 and NOD2 signaling, SHIP-1 competes with RIP2 for the binding to XIAP. Actually, we do not know why the interaction between SHIP-1 and XIAP is only detected upon NOD1 and NOD2 activation. Do MDP or Tri-DAP treatments induce a posttranslational or a conformational modification of XIAP that enables its interaction with SHIP-1 or *vis-versa*?

Strikingly, we observed that upon treatment with MDP, SHIP-1 and XIAP are recruited to the membrane fraction. Indeed, it has been previously described that the NODsome forms and signals from the plasma membrane [43], [44], [50]. This membrane location is crucial. Hence, it was demonstrated that mutated forms of NOD1 and NOD2, which failed to activate NF-κB, also failed to locate at the plasma membrane [44], [50], [51]. Thenceforward, it is speculated that plasma membrane location reflects the activation status of NOD2 and NOD1 proteins [50]. Furthermore, oligomerized NOD1 or NOD2 proteins constituted a platform enabling the recruitment of RIP2 and other adaptor molecules to the plasma membrane, thereby triggering NF-κB signaling. Indeed, enforced membrane localization of RIP2 is sufficient to induce NF-κB activation, showing that the optimal RIP2 activity relies on its subcellular location [43]. Wherefore, the translocation of XIAP to the membrane fraction was not surprising, since several components of the NODosome are recruited in this subcellular fraction. The recruitment of SHIP-1 to the plasma membrane during NOD2 stimulation reinforces our hypothesis about the role of SHIP-1 in NOD2 signaling. Interestingly, SHIP-1 is known to be recruited to the plasma membrane *via* its PRD domain. Indeed, SHIP-1ΔPRD protein is unable to locate at the plasma membrane [52], [53]. Strikingly, it seems that SHIP-1 decreases the membrane recruitment of XIAP upon MDP stimulation. As SHIP-1 also downregulates the interaction between XIAP and RIP2, we suggest the following model to explain our observations: after NOD2 engagement, XIAP is recruited to the plasma membrane *via* its interaction with RIP2. As XIAP was reported to interact with the TAK1/TAB1/2 complex, this interaction facilitates the IKK complex activation and thereby the NF-κB signaling. After long-time exposure to MDP (2 to 4 h), SHIP-1 interacts with XIAP. As SHIP-1 interacts also with the BIR2 domain of XIAP, it displaces XIAP from its interaction with RIP2. Thenceforward, XIAP is removed into the cytosol. Accordingly, the IKK activation decreases, leading to a decreased cytokines secretion.

In this report, we have demonstrated for the first time that SHIP-1 is a negative regulator of both NOD1 and NOD2 signaling pathway. We have highlighted that SHIP-1 exerts its inhibitory capacity by interacting with XIAP, thereby altering its interaction with RIP2 and decreasing NF-κB activation. Understanding precise mechanism of NOD1 and NOD2 regulation by SHIP-1 would help to develop new therapeutics in the field of inflammatory disorders.

## Materials and Methods

### Cell Lines and Culture

Human embryonic kidney-293T cell line (HEK-293T, purchased form the ATTC) and GNV cells (see stable cell lines section) were cultured in Dulbecco’s Modified Eagle’s medium (Lonza, Belgium) supplemented with 10% fetal bovine serum, 1% glutamine (Gibco®, Invitrogen) and 1% antibiotics (streptomycin and penicillin). For GNV cells medium is supplemented with blasticidin S at 15 µg/mL (Invitrogen). Monocytic cell line THP1 (purchased from the ATCC), THP1-Xblue (purchased form InvivoGen) and THP1 stably expressing shRNA (see stable cell lines section) were cultured in RPMI medium (Lonza, Belgium) supplemented with 10% fetal bovine serum, 1% glutamine (Gibco®, Invitrogen) and 1% antibiotics (streptomycin and penicillin). THP1-Xblue cells are selected by treatment with zeocin at 200 µg/mL (Invitrogen). Selection of THP1 cells expressing shCTRL and shSHIP-1 was made by culturing cells with puromycin at 1 µg/mL (Sigma-Aldrich).

### Stable Cell Lines

GNV cells were obtained by stable lentiviral transduction of HEK293 cells (purchase from ATTC, catalog number CRL-1573) with a GFP-NOD2-V5 construct (see plasmids section). The lentiviral particles were generated by transducing Lenti-X™ 293T cells (Clontech) with a pSPAX2 (Addgene) and a VSV-G encoding vector (Addgene) along with a GFP-NOD2-V5 encoding plasmid. 24 h post transfection, viral supernatants were ultracentrifuged at 50,000 × *g* for 2 h at 4°C. The pellet was resuspended in 25 µL of PBS medium (Lonza, Belgium) and incubated over night at 4°C. The next day, viral concentration was determined and HEK293 were transduced with an MOI of 10. Selection of transduced cell was achieved by adding blasticidin S at 15 µg/mL (InvivoGen) to the cell culture medium. The transduced HEK293 cells expressing a NOD2 intermediate level and allowing a MDP inducible NF-κB activation were isolated by fluorescence-activated cell sorting and called GNV cells for HEK293 cells stably expressing GFP-NOD2-V5. THP1-Xblue cells (generated by InvivoGen – catalog code thpx-sp) were a kind gift from Thomas Kufer (Institute for Medical Microbiology, Immunology and Hygiene, University of Cologne, Cologne, Germany). THP1 expressing shCTRL and shSHIP-1 were generated via lentiviral transduction (by the GIGA-viral vector platform). The lentiviral particles were generated by transducing Lenti-X™ 293T cells (Clontech) with a pSPAX2 (Addgene), a pCG-H_24 [54], and a pCG-F_30 encoding vectors [54] along with a non-target shRNA control (Sigma-Aldrich) or a human SHIP-1 shRNA (Sigma-Aldrich, sequence: 5′-CCGGAATTGCGTTTACACTTACAGCTCGAGCTGTAAGTGTAAACGCAATTCTTTTTG-3′) encoding vectors, respectively. 48 h post transfection, viral supernatants were ultracentrifuged at 50,000×*g* for 2 h at 4°C. The pellet was resuspended in 100 µL of GMP Serum-free Stem Cell Growth Medium (CellGro), viral concentration was determined and THP-1 cells (purchased from ATCC, catalog number TIB-202) were transduced with an MOI of 10. Selection of transduced cells was achieved by adding puromycin (3 µg/mL) to the cell culture medium.

### Antibodies and Reagents

Muramyl dipeptide and phorbol 12-myristate 13-acetate (PMA) were purchased from Sigma-Aldrich and tumor necrosis factor-α (TNF- α, #300-01A) was obtained from PeproTech EC (UK). Tri-DAP and D-D-MDP (MDP control) were purchased from InvivoGen. Anti-SHIP-1 (P1C1), anti-RIP2 (H300) and anti-NF-κB p100/p52 was from Santa Cruz, Anti-XIAP was from BD Transduction Laboratories, anti-GFP was from Roche, anti-Calnexin was from Abcam, anti-HA from Sigma-Aldrich, anti-p-IκBα Ser32/36, anti-myc and anti-p-IKKβ were from Cell Signaling.

### Plasmids

pcDNA 3.1 Myc-SHIP-1 was generated by subcloning SHIP-1 coding sequence into pcDNA3.1 using the pcDNA™3.1 Directional TOPO® Expression Kit (Invitrogen). Gateway expressing vector HA-PRD and Myc-ΔPRD were generated by subcloning the corresponding SHIP-1 coding sequence into pDONR233 via BP clonase reaction and subsequent transfert into pDest475 or pCS3MTdest via a LR clonase reaction (Invitrogen). Gateway expressing vector YFP-XIAP was generated by transferring XIAP coding sequence from pDONR223 XIAP (ORFeome 5.1) into pDest491 via LR clonase reaction. pDest491, pDest475 and pDONR223 were a kind gift from Jean-Claude Twizere (Laboratory of Protein Signaling and Interactions (PSI), GIGA-R, Liège, Belgium). pCS3MTdest was a kind gift from Josef Martial (Molecular biology and genetic engineering unit, GIGA-R, Liège, Belgium). pcDNA FLAG-XIAP was a kind gift from Sylvain Latour (Laboratoire du Développement Normal et Pathologique du Système Immunitaire, Inserm U768, Paris). Expression plasmids encoding wild-type NOD2 and HA-tagged were a kind gift from G. Nunez (University of Michigan, Medical School & Comprehensive Cancer Center, Ann Harbor, MI). GFP-NOD2 was generated by PCR using the GFP Fusion TOPO® TA expression kit (Invitrogen) from HA-NOD2.

### Yeast Two-hybrid Screen

pDB-SHIP-1, pDB-SH2 and pBD-PRD vectors encoding SHIP-1 WT, SH2 domain, or PRD domain, respectively, were cloned into Gateway®pDEST™ 32 (Invitrogen) by LR clonase reaction (Invitrogen) and transformed into *Saccharomyces cerevisiae* Y8930 strain [37]. The human hORFeome 5.1 cloned into the Gateway®pDEST™ 22 (Invitrogen) and fused to the Gal4 activation domain was provided into *Saccharomyces cerevisiae* Y8800 strain [37]. After mating of the two yeast strains, cotransformants were plated on media lacking tryptophan, leucine and histidine (for detection of weak interaction) and on media lacking tryptophan, leucine and adenine (for detection of strong interaction), three days later, growing colonies, scored as positive for interaction, were isolated; the interested genes were amplified by PCR and identified by sequencing. Y2H retesting was achieved by pair-wise mating between pDB-SHIP-1 WT, pDB-SH2 and pDB-PRD with isolated pAD-interaction partner candidate. The cotransformants were selected on media lacking tryptophan, leucine and histidine and on media lacking tryptophan, leucine and adenine. The growing colonies were identified.

### Protein Extraction

Cells were washed with cold PBS, scraped and centrifuged. The cells were lysed in XIAP lysis buffer (20 mM Tris-HCl pH 7.5, 135 mM NaCl, 1 mM EDTA, 0.5% NP-40 and 10% glycerol), incubated 10 min on ice and centrifuged 15 min at 14,000 × *g* at 4°C. Protein concentrations were measured with the Bio-Rad protein assay, the extracts were subsequently subjected to SDS-PAGE, transferred onto PVDF membrane and incubated with various antibodies as specifically indicated.

### Cellular Fractionation

Cells were harvested, lysed in a cytosolic lysis buffer (10 mM Tris-HCl pH 7.9, 0.34 M sucrose, 3 mM CaCl_2_, 0,1 mM EDTA, 2 mM MgCH_3_COO, 0.5% NP40), incubated on iced for 15 min and centrifuged at 100×*g* for 10 min at 4°C. The pellet was washed two times with the cytosolic lysis buffer before being homogenized in the nuclear lysis buffer (10 mM Tris-HCl ph7.9, 50 mM NaCl, 5 mM MgCl_2_, 5 mM EDTA, 5% glycerol, 0,1% NP40, 0,1% Triton X-100 and 0,1% Tween 20). This fraction constituted the nuclear extract. The supernatant of the first centrifugation at 100 × *g* was subsequently ultracentrifuged at 35 000×*g* for 30 min. The supernatant was again ultracentrifugated at 100 000 × *g* for 1 h. The supernatant of these second ultracentifugation was harvested and constituted the cytosolic fraction. The pellet of the first ultracentrifugation at 35 000 × *g* was washed two times with the cytosolic buffer before being homogenized in this cytosolic buffer. This extract constituted the membrane fraction. Equal among of each fraction (cytosolic, membrane and nuclear) were subjected to SDS-PAGE.

### Immunoprecipitation

For immunoprecipitation (IP), cells were lysed in XIAP lysis buffer (20 mM Tris-HCl pH 7.5, 135 mM NaCl, 1 mM EDTA, 0.5% NP-40 and 10% glycerol). Clarified lysates were incubated with 4 µg of anti-GFP (Sigma-Aldrich) or 5 µg of anti-SHIP-1 (Santa Cruz) or 4 µg of anti-XIAP (BD Transduction Laboratories) for 2 h at 4°C. 30 µL of A-agarose beads (Thermo-Fisher) were added to the mix. After 2 h of incubation beads-linked immune complex were washed three times with XIAP lysis buffer, boiled in TR buffer 2X (10 mM Tris-HCl pH 6.8, 1% SDS, 25% glycerol, 0.1 mM β-mercaptoethanol, 0.003% bromophenol blue) and analyzed by SDS-PAGE. In order to avoid cross reaction with the heavy chain of antibody used for immunoprecipitation, XIAP and HA-PRD construct were detected with an anti-light chain specific HRP-linked secondary antibody from Jackson Immunoresearch Europe Ltd. RIP2 immunoprecipitation were performed according to the same protocol except for the lysis buffer which contain SDS to solubilise membrane fraction (10 mM Hepes-KOH pH 8, 150 mM NaCl, 1% NP-40, 0,1% SDS, 0,5% sodium deoxycholate).

### siRNA Transfection

THP1-Xblue cells were differentiated in macrophages by treatment with 12.5 ng of PMA (Sigma-Aldrich) for 48 h. The cells were subsequently transfected with 0.5 µM of a siRNA targeting SHIP-1 (sequence: 5′-GCUAAGUGCUUUACGAACA-3′, see ref [36]) or XIAP (QIAGEN) or a non-target siRNA (Ambion) using HiPerfect transfection reagent (QIAGEN). 48 h post transfection the cells were treated with MDP or Tri-DAP during 16 h before the colorimetric enzyme assay with the QuantiBlue™ (Invitrogen) or during 8 h before the ELISA assay (Pierce). Alternatively, for protein and RNA extractions, the cells were treated, 48 h post transfection, for indicated periods with MDP (100 µg/mL).

### GST-pull Down Assay

GST-XIAP WT, -BIR1, -BIR2, -BIR3 and -BIR3-UBA were a kind gift of Dr. Sylvain Latour (Inserm U768, Paris). Bacterially expressed GST-XIAP fusion protein were purified on glutathione agarose beads and stored at –80°C. HEK-293T were transfected with pDest 475 HA-PRD or with the pcDNA 3.1 SHIP-1 WT. 24 h after transfection, cells were lysed in LB-glycerol buffer (50 mM Tris-HCl pH 8, 150 mM NaCl, 2 mM EDTA, 1% NP-40, 10% glycerol), 700 µg of protein extract were precleared with 200 µg of recombinant GST protein immobilized on glutathione agarose beads for 1 h at 4°C. The precleared lysates were next incubated with the different recombinant GST-XIAP constructs (or with GST alone as negative control) for 2 h at 4°C. The beads were then washed three times with LB buffer and the pulled down proteins were eluted with SDS loading buffer and subjected to SDS-PAGE. Positive control was achieved by incubating HEK293T lysate with cytochrome C (Sigma-Aldrich, C-7752) and dATP (Invitrogen) for 30 min at 37°C to induce a cleavage of caspase-3. This lysate was next precleared and subsequently incubated with 200 µg of GST-BIR2 construct.

### Colorimetric Enzyme Assay with QuantiBlue™

20 µL of THP1-Xblue cell supernatant was mixed with 200 µL of QuantiBlue solution (InvivoGen) and incubated at 37°C for 2 h. Absorbance was measured at 630 nm.

### IL-8 ELISA

THP1 cells were transfected with a siRNA targeting SHIP-1 as previously described. 48 h after transfection, cells were treated with MDP (100 µg/mL) during 4 h or 8 h and cell supernatants were harvested. IL-8 protein concentration was determined by IL-8 ELISA according to the manufactured protocol (Pierce).

### Luciferase Assay

HEK293T or GNV cells were transfected with lipofectamine™2000 reagent (Invitrogen) with indicated plasmid(s) (see figures) and a luciferase reporter plasmid under the control of a κB-dependent promoter. GNV cells were treated for 16 h with 1 µg of MDP or left untreated. 36 h after transfection cells were lysed and assayed for luciferase activity with a Luciferase Reporter Gene Assay according to manufacturer protocol (Roche). The κB-dependent luciferase activity was normalized to protein concentration measured by Bio-Rad protein assay.

### Quantitative Real Time Reverse Transcription-PCR

Total RNA samples were isolated with the RNeasy kit (QIAgen) according to manufacturer protocol. 1 µg of RNA was submitted to reverse transcription with the moloney murine leukemia virus reverse transcriptase (Invitrogen). cDNA obtained for each samples were submitted to qRT-PCR using teh SYBR green Master mix method (Eurogentec, Liège, Belgium) in teh ABI 7000 Sequence Detection System (Applied Biosystem). The results were normalized with the β-2-microglobulin transcript. The primers used to analyze the different transcripts were designed with the software Primer Express™ (Applied Biosystems): *il-8*, FW: 5′-GAAGGAACCATCTCACTGTGTGTAA-3′ and RV: 5′-ATCAGGAAGGCTGCCAAGAG-3′ *; il-6*, FW: 5′-CCAGGAGCCCAGCTATGAAC-3′ and RV: 5′-CCCAGGGAGAAGGCAACTG-3′; *tnf-*α, FW: 5′-GGAGAAGGGTGACCGACTCA-3′ and RV: 5′-TGCCCAGACTCGGCAAAG-3′; *B2M*, FW : 5′-GAGTATGCCTGCCGTGTG-3′ and RV: 5′-AATCCAAATGCGGCATCT-3′.

## Supporting Information

Figure S1 **SHIP-1 decreases NOD1-induced NF-κB activation. A,B.** THP1-Xblue cells were transfected with a siRNA targeting SHIP-1 (siSHIP-1) or with a non-target siRNA (siCTRL). 48 h after transfection, cells were treated with Tri-DAP (75 µg/mL) or with MDP (100 µg/mL) for indicated periods. RNAs were extracted and *tnf-α* and *il-6* mRNA levels were measured by quantitative real-time PCR (Mean ± S.D (n = 3), *p<0.0001).
(TIF)

Figure S2 **XIAP is an essential and specific intermediate of NOD1-induced NF-κB activation. A,B.** THP1-Xblue cells were transfected with a siRNA targeting XIAP (siXIAP) or with a non-target siRNA (siCTRL). 48 h after transfection, cells were treated with Tri-DAP (75 µg/mL) or with MDP (100 µg/mL) for indicated periods. RNAs were extracted and *tnf-α* and *il-6* mRNA levels were measured by quantitative real-time PCR (Mean ± S.D (n = 3), *p<0.0001).
(TIF)
