# Supplementary figures and images for: The Inositol Phosphatase SHIP-1 Inhibits NOD2-Induced NF-κB Activation by Disturbing the Interaction of XIAP with RIP2
Source: PLoS One. 2012 Jul 17;7(7):e41005. doi: 10.1371/journal.pone.0041005 (PMC3398883; doi:10.1371/journal.pone.0041005)

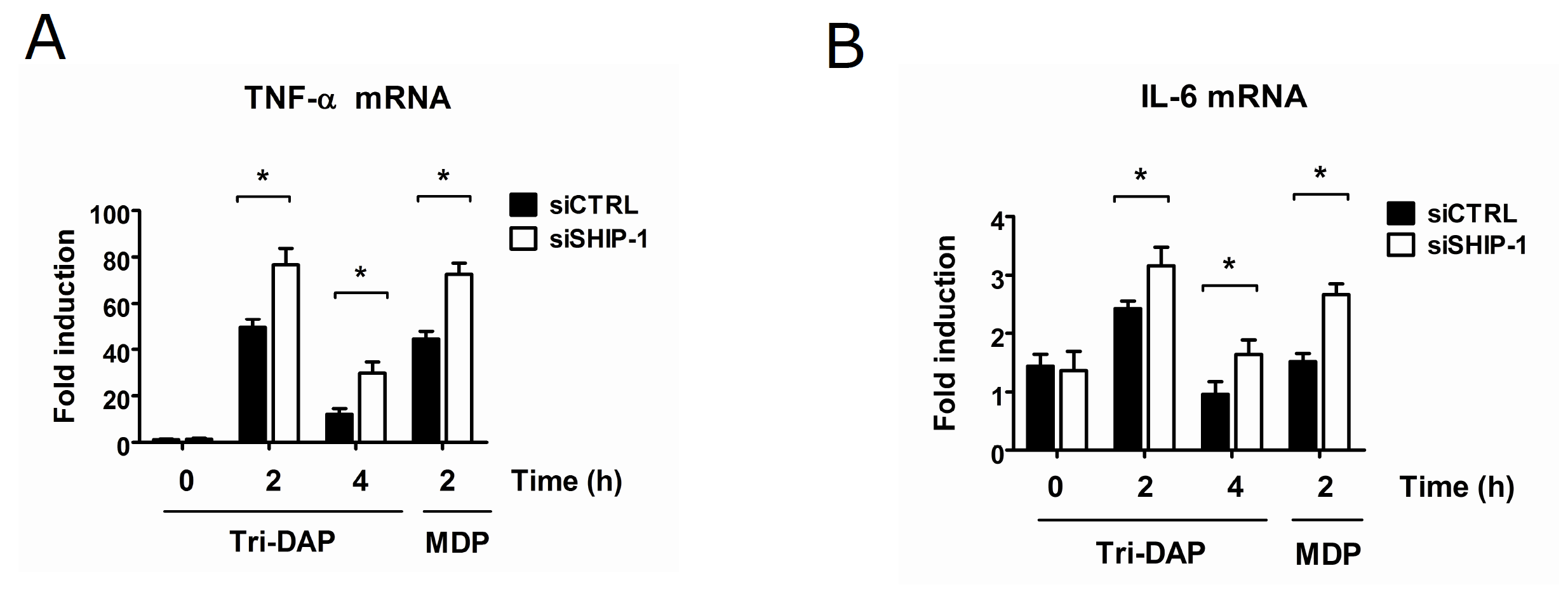

Supplement: Figure S1 — SHIP-1 decreases NOD1-induced NF-κB activation. A,B. THP1-Xblue cells were transfected with a siRNA targeting SHIP-1 (siSHIP-1) or with a non-target siRNA (siCTRL). 48 h after transfection, cells were treated with Tri-DAP (75 µg/mL) or with MDP (100 µg/mL) for indicated periods. RNAs were extracted and tnf-α and il-6 mRNA levels were measured by quantitative real-time PCR (Mean ± S.D (n = 3), *p<0.0001). (TIF) [file pone.0041005.s001.tif]

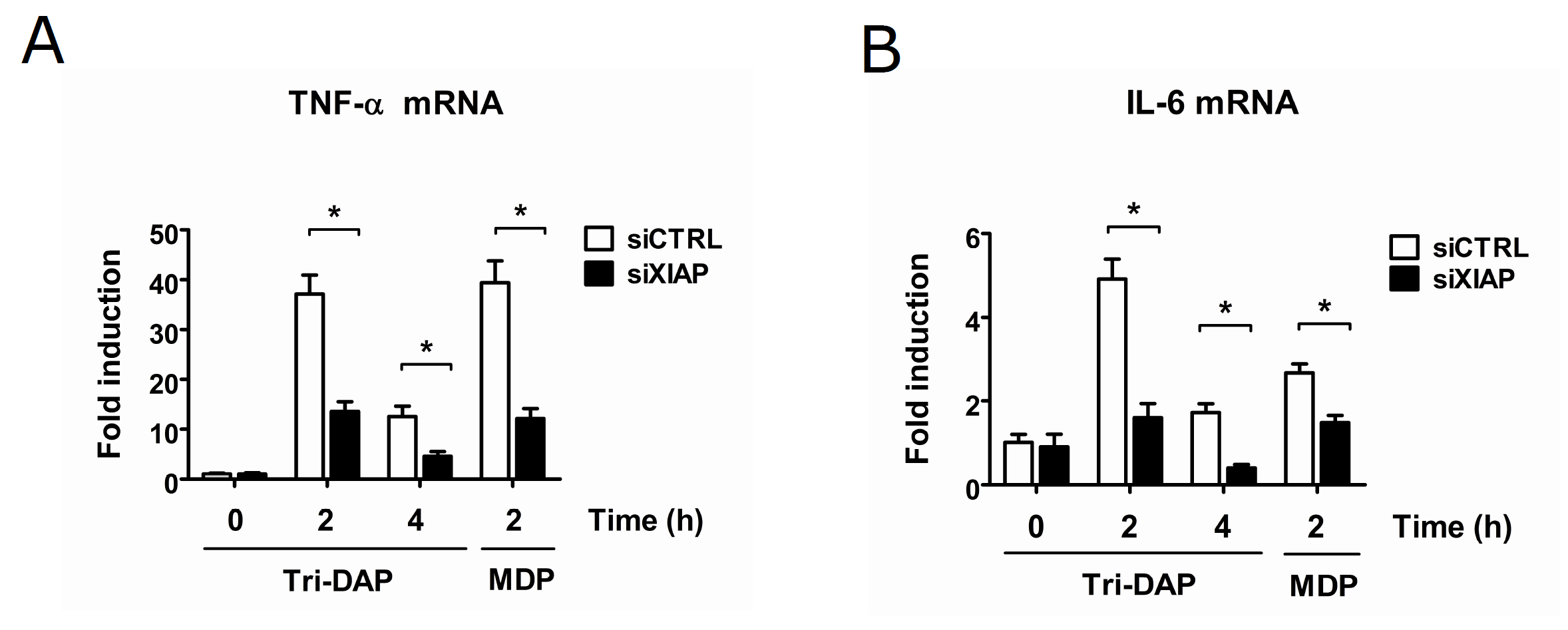

Supplement: Figure S2 — XIAP is an essential and specific intermediate of NOD1-induced NF-κB activation. A,B. THP1-Xblue cells were transfected with a siRNA targeting XIAP (siXIAP) or with a non-target siRNA (siCTRL). 48 h after transfection, cells were treated with Tri-DAP (75 µg/mL) or with MDP (100 µg/mL) for indicated periods. RNAs were extracted and tnf-α and il-6 mRNA levels were measured by quantitative real-time PCR (Mean ± S.D (n = 3), *p<0.0001). (TIF) [file pone.0041005.s002.tif]
